# Exploring Social Support Systems and Experiences of Older Women in Tede Community, Oyo State, Nigeria.

**DOI:** 10.1093/geroni/igab046.3312

**Published:** 2021-12-17

**Authors:** Kafayat Mahmoud

**Affiliations:** University of Kansas, Lawrence, Kansas, United States

## Abstract

Rural communities in Nigeria are rapidly aging due to the massive movement of young adults to the cities, especially after marriage and/or in search of employment. This has adversely affected the social supports for older persons. The current study explored the experiences of older adults living in rural communities in Southwestern Nigeria. The study adopted an exploratory qualitative research design, in-depth interview techniques were adopted, and results were analyzed using thematic analysis. For this pilot study, conducted in Tede community in Oyo state in Nigeria, 10 older women, aged 70+ years were purposively selected for the study. The fact that all participants were women was due to the fact that older persons found in the community were mostly women. Consonant with previous research, this pilot study found that there was limited formal and informal support systems for older women in the community. The study additionally revealed that older women expressed feelings of abandonment by adult children, having insufficient funds, as well as inability to access health care. Consequently, these women resorted to alternative means to fend for themselves. For instance, despite having functional limitations, older women in the community would engage in physically demanding tasks such as going into the bushes to cut and gather firewood to sell, as well as engaging in other forms of petty trading, while others resorted to begging for alms for sustenance. This pilot study highlights the experience of poverty among older women and the need for more structural interventions for older persons in Nigeria.

